# Leptospirosis y rickettsiosis, reto diagnóstico para el síndrome febril en zonas endémicas

**DOI:** 10.7705/biomedica.5598

**Published:** 2021-06-15

**Authors:** René Ramírez-García, Juan Carlos Quintero, Aixa Paola Rosado, Margarita Arboleda, Víctor Alejandro González, Piedad Agudelo-Flórez

**Affiliations:** 1 Grupo de Investigación en Ciencias Básicas, Escuela de Graduados, Universidad CES, Medellín, Colombia Universidad CES Universidad CES Medellín Colombia; 2 Grupo de Investigación en Ciencias Veterinarias “Centauro”, Facultad de Ciencias Agrarias, Universidad de Antioquia, Medellín, Colombia Universidad de Antioquia Facultad de Ciencias Agrarias Universidad de Antioquia Medellín Colombia; 3 Hospital Antonio Roldán Betancur, IPS Universitaria, Apartadó, Colombia Hospital Antonio Roldán Betancur IPS Universitaria Apartadó Colombia; 4 Grupo de Investigación en Medicina Tropical, Instituto Colombiano de Medicina Tropical - ICMT- CES, Apartadó, Colombia Universidad CES ICMT CES Apartadó Colombia

**Keywords:** Leptospirosis/diagnóstico, infecciones por Rickettsiaceae/diagnóstico, fiebre, hemorragia, zoonosis, Leptospirosis/diagnosis, Rickettsiaceae infections/diagnosis, fever, hemorrhage, zoonosis

## Abstract

Se presenta el caso de un hombre de 50 años de edad proveniente de la región de Urabá, Colombia, con una infección mixta por *Rickettsia rickettsii* y *Leptospira interrogans* serovar Copenhageni ST78, y pruebas negativas para malaria y dengue.

El paciente presentó un síndrome febril que no mejoró con el tratamiento antibiótico sistémico y, finalmente, falleció en la unidad de cuidados intensivos. El diagnóstico *post mortem* se hizo mediante tipificación molecular de los dos agentes etiológicos. En la inspección del domicilio del paciente, se encontró un ejemplar de *Rattus rattus* infectado con *L. interrogans* del mismo serovar detectado en él. No se encontraron garrapatas en los animales domésticos que habitaban con el paciente.

Se reporta una infección mixta con síntomas clínicos progresivos y fatales en un paciente con antecedentes laborales de riesgo en una zona endémica para enfermedades tropicales, lo que obliga a tener presente la posibilidad de infecciones simultáneas en personas procedentes de áreas endémicas que consulten reiteradamente por síndrome febril sin resolución y tengan riesgo laboral relacionado con actividades agrícolas.

## Abstract (en)

This is the case of a 50-year-old male from the region of Urabá, Colombia, with a mixed infection by *Rickettsia rickettsii* and *Leptospira interrogans* serovar Copenhageni ST78 and negative test for malaria and dengue fever.

The patient presented with febrile syndrome and was unresponsive to systemic antibiotic treatment, who finally died in the intensive care unit. We established the postmortem diagnosis through molecular typification of the two etiological agents. In the inspection at the patient’s home, we found a *Rattus rattus* specimen infected with *L. interrogans* of the same serovar found in him. We found no ticks parasitizing the domestic animals cohabitating with the patient.

This case of a mixed infection with progressive and fatal symptoms in a patient with occupational risk in a tropical disease endemic zone highlights the importance of considering the potential presentation of simultaneous etiologies in patients with multiple medical visits for unresolved febrile syndromes associated with risky exposure during agricultural activities.

Las enfermedades tropicales representan un problema de salud pública en la región de Urabá. Del 20 al 30% de quienes las contraen ingresan a las unidades de cuidados intensivos por presentar diarreas, malaria, dengue, fiebre tifoidea, enfermedad por rickettsias y leptospirosis (1). El establecer la causa del síndrome febril humano en las regiones tropicales constituye un reto para los médicos debido a que los cuadros clínicos de muchas de estas enfermedades son similares: fiebre, erupciones cutáneas, trombocitopenia, función hepática levemente alterada, insuficiencia respiratoria, renal, hepática o circulatoria con alteración del estado mental, convulsiones y coagulopatía (2).

Se sabe que el diagnóstico tardío del síndrome febril tiene implicaciones potencialmente fatales, pero sus manifestaciones inespecíficas, sumadas a la posible presencia simultánea de varios agentes prevalentes en zonas tropicales, hacen que el reto del diagnóstico adquiera dimensiones desconocidas (1). En estas zonas -donde confluyen múltiples agentes infecciosos de naturaleza viral, parasitaria y bacteriana- la probabilidad de que un humano tenga una infección mixta es muy alta. La leptospirosis y la fiebre manchada son enfermedades infecciosas zoonóticas que se manifiestan clínicamente como síndromes febriles agudos y que pueden evolucionar hacia cuadros clínicos graves (3,4).

En las instituciones de salud no se incluyen de manera rutinaria las pruebas de diagnóstico molecular y muchos de los métodos diagnósticos empleados en los pacientes con síndrome febril agudo se centran en la malaria y el dengue, a pesar de que en los países tropicales se ha demostrado la circulación de otros agentes infecciosos relacionados con dicho síndrome (5,6).

En varios países del mundo se han descrito casos de rickettsiosis o leptospirosis en humanos con un desenlace fatal (7-9) y, en muchos de tales casos, confluyen dos situaciones: el diagnóstico tardío y los tratamientos inoportunos.

En este reporte se presenta el caso de un paciente con infección mixta causada por dos agentes potencialmente letales, cuyo control eficaz se logra con un diagnóstico oportuno y una intervención terapéutica específica y temprana. El conocimiento de la evolución clínica de este paciente proveniente de una zona endémica para otras enfermedades tropicales y zoonóticas con síndrome febril no malárico diagnosticado *post mortem* causado por una infección mixta, es de interés para lograr la intervención terapéutica oportuna en casos similares y evitar así posibles desenlaces fatales.

## Presentación del caso

Se trata de un hombre de 50 años de edad sin antecedentes de enfermedad que trabajaba en oficios varios en una finca bananera de la vereda Zungo, área rural del municipio de Carepa (Antioquia, Colombia), zona endémica para enfermedades icterohemorrágicas como el dengue, la fiebre de chikunguña, la malaria, la leptospirosis y la rickettsiosis.

En su primera consulta, el paciente refirió que la enfermedad había sido de inicio insidioso y que había presentado fiebre no cuantificada asociada con dolor osteomuscular generalizado, malestar y escalofríos. Como antecedente, relataba haber estado expuesto a alcantarillados donde había muchos roedores. En el examen físico, sus signos vitales fueron normales y no presentó ningún hallazgo de importancia. Le diagnosticaron una infección viral inespecífica que se podía tratar ambulatoriamente y, después de los exámenes paraclínicos, fue dado de alta (cuadro 1).

**Cuadro 1.** Examen de hematología del caso clínico de leptospirosis-rickettsiosis fatal

Parámetro	Valor	Valor normal*
Hemoglobina (g/dl)	14,1	14 - 17,5
Hematocrito (%)	43,60	40- 52
Eritrocitos (por mm^3^)	4,77 x 10^6^	3,38 - 6,76 x 10^6^
Volumen corpuscular medio (fl)	91,4	80 - 94
Hemoglobina corpuscular media (pg)	29,6	
Concentración de hemoglobina corpuscular media (g/dl)	32,3	33 - 37
Distribución eritrocitaria (%)	13,8	11,5 - 14,5
Leucocitos totales (por mm^3^)	7,69 x 10^3^	4,8 - 11 x 10^3^
Neutrófilos (por mm^3^)	4,50 x 10^3^	2 - 7,4 x 10^3^
Linfocitos (por mm^3^)	2,36 x 10^3^	0,7 - 4,5 x 10^3^
Eosinófilos (por mm^3^)	0,14 x 10^3^	0 - 0,07
Monocitos (por mm^3^)	0,65 x 10^3^	0,1 - 1 x 10^3^
Basófilos (por mm^3^)	0,04 x 10^3^	0 - 0,2 x 10^3^
Plaquetas (por mm^3^)	330 x 10^3^	150 - 500 x 10^3^
Bandas (%)	0,10	0 - 3

* Los valores normales se establecen según los parámetros del laboratorio que realiza la prueba.

Nueve días después de la consulta inicial, el paciente regresó al servicio de salud con los mismos síntomas y, además, con signos de irritación urinaria (disuria, polaquiuria) y coluria. Sus signos vitales eran normales y presentó sensibilidad en la fosa renal izquierda en el examen físico; se decidió darlo de alta después de la revisión ambulatoria de los exámenes de laboratorio (uroanálisis y hemoparásitos en sangre) y un tratamiento sintomático similar al inicial (acetaminofén). 

Trece días después, el paciente consultó nuevamente por síntomas de tres días de evolución consistentes en cefalea (que no había referido anteriormente), poliartralgias y escalofríos, y fiebre de 38,9 °C. Se le practicaron los exámenes de laboratorio ya ordenados, y los de función renal sugirieron daño tubular y glomerular (cuadro 2).

**Cuadro 2:** Resultados del análisis citoquímico de orina de seguimiento del caso clínico fatal de leptospirosis-rickettsiosis

Parámetros	Resultado
Color	Amarillo
Apariencia	Turbio
Glucosa	Normal
Bilirrubinas	17 (μmol/L)
Cetonas	Negativas
Densidad relativa	1.023
Sangre	150 células
pH	6
Proteínas	Negativas
Urobilinógeno	Normal
Nitritos	Negativos
Leucocitos	Negativos
Eritrocitos crenados	>50 por campo
Cristales de urato amónico	Abundantes
Bacterias	Escasas

Como el paciente estaba siendo atendido en consulta externa, dichos exámenes fueron revisados tan solo en la siguiente consulta; en ese momento, el médico tratante determinó que el paciente presentaba un cuadro clínico sugestivo de infección de las vías urinarias con compromiso renal sugestivo de daño túbulo-glomerular. Se le dio de alta y se le prescribió nuevamente acetaminofén.

Al no presentar mejoría, el paciente regresó dos días después; además de su compleja sintomatología, esta vez presentaba dolor hipogástrico evidente en el examen físico, pero sin irritación peritoneal, por lo que le recetaron gentamicina, nitrofurantoína y ácido ascórbico, así como los antiinflamatorios no esteroideos y el acetaminofén que ya venía tomando. Lo dieron de alta con incapacidad laboral de un día.

Dos días después, el paciente consultó al servicio de urgencias de un centro de alta complejidad, donde ingresó en malas condiciones generales: taquicárdico, con frecuencia cardiaca de 117 por minuto; hipotenso, con presión arterial de 86/50 mm Hg, frecuencia respiratoria de 20 por minuto, y exantema en el abdomen, por lo que se inició administración moderada de líquidos intravenosos (bolo de 300 ml más infusión a 150 ml/hora), se le tomaron exámenes paraclínicos (cuadro 3) y se intensificó el tratamiento antimicrobiano con ceftriaxona, con base en un diagnóstico de sepsis de origen aún por esclarecer.

**Cuadro 3:** Resultados de exámenes paraclínicos y gases sanguíneos antes de la muerte del paciente con leptospirosis-rickettsiosis

Parámetro	Valor	Valor normal*
Hemoglobina (g/dl)	15	14 - 17,5
Hematocrito (%)	43,90	40 - 52
Leucocitos (por mm^3^)	18,92 x 10^3^	4,8 -11 x 10^3^
Neutrófilos (por mm^3^)	17,74 x 10^3^	2 - 7,4 x 10^3^
Linfocitos (por mm^3^)	1,9 x 10	0,7 - 4,5 x 10^3^
Plaquetas (por mm^3^)	28 x 10^3^	150 - 500 x 10^3^
Proteína C reactiva (mg/dl)	29,2	0 - 5
AST (U/L)	298	0 - 34
ALT (U/L)	128	10 - 49
Sodio sérico (mEq/L)	129	135 - 145
Potasio sérico (mEq/L)	3,47	3,5 - 5,0
Cloro sérico (mEq/L)	99	100 - 106
Calcio sérico ionizado (mmol/L)	1,02	1,0 - 1,3
Gases arteriales		
pH	7,393	7,37 - 7,45
pCO_2_ (mm Hg)	15,8	36 - 45
pO_2_ (mm Hg)	86	90 - 110
HCO_3_ (mmol/L)	9,4	24 - 34
PaO_2_/FiO_2_	410	
Exceso de base (mEq/L)	-12	2,3 - -2,3
Lactato (mmol/L)	9,7	1,0 - 1,5

* Los valores normales se establecen según los parámetros del laboratorio que realiza la prueba.

Dado el rápido deterioro del paciente al no responder al tratamiento descrito, así como la aparición de trombocitopenia grave en el cuadro sindromático, fue internado en la unidad de cuidados intensivos donde sus signos vitales empeoraron progresivamente, con mayor trabajo respiratorio y taquipnea en avance, orientación fluctuante, falla multiorgánica, petequias e ictericia.

En los rayos X de tórax se evidenciaron signos de sobrecarga hídrica con un patrón intersticial; el estado clínico del paciente continuó deteriorándose con mayor inestabilidad hemodinámica, hipoperfusión cerebral con alteración del estado de conciencia e incoherencia fluctuante, lo que sugería la presencia de un delirio de origen multifactorial; además, presentó falla respiratoria, por lo cual requirió vasopresores y asistencia respiratoria mecánica.

Ante la sospecha de fiebre hemorrágica y los resultados negativos de las pruebas para arbovirus, se solicitó la confirmación serológica de *Leptospira* spp. y una prueba para rickettsiosis.

Dada la sospecha de leptospirosis grave, el tratamiento se modificó para administrarle penicilina sódica intravenosa y doxiciclina, y se complementó con una reposición plaquetaria por aféresis para tratar el síndrome hemorrágico posiblemente infeccioso.

Al cabo de 24 horas en la unidad de cuidados intensivos, el paciente falleció por falla multiorgánica, acidosis grave e inestabilidad respiratoria.

**Figura 1:**
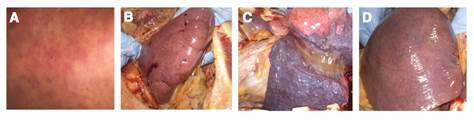
Fotografías de hallazgos *post mortem.* Paciente masculino 50 años de edad. Diagnóstico positivo para leptospira y rickettsiosis. **A.** Erupción cutánea con lesiones hemorrágicas. **B.** Riñón con presencia de zonas hemorrágicas generalizadas. **C.** Pulmón con evidencia de alteración pulmonar hemorrágica. **D.** Hígado megálico con evidencia de abundantes áreas hemorrágicas.

En la autopsia, se halló exantema petequial generalizado, estigmas de hemorragia parenquimatosa petequial en hígado, bazo y riñones, signos de extensa hemorragia pulmonar intraparenquimatosa, hepatoesplenomegalia de aspecto francamente congestivo y hallazgos sugestivos de encefalitis (figura 1).

En el examen microscópico se encontró: cambios hipóxico-isquémicos en la corteza cerebral; tenue infiltrado linfoplasmocitario y alteraciones en leptomeninges, sugestivos de meningitis aguda temprana; daño pulmonar alveolar difuso en fase exudativa fibrinoide, con microhemorragia reciente, edema y atelectasia; hepatitis crónica moderada en estado de fibrosis temprana; necrosis tubular renal aguda focal y nefritis intersticial aguda, y congestión cardiaca difusa sin proceso inflamatorio.

Se tomaron múltiples muestras tisulares para el estudio de diagnóstico molecular y, también, se realizó una visita al domicilio del paciente fallecido durante la cual se capturaron roedores (*Rattus rattus*), pero no se encontraron garrapatas en los animales domésticos revisados. Las muestras obtenidas de las necropsias de los roedores también se usaron para el diagnóstico molecular.

## Diagnóstico molecular

Se hizo la detección molecular de los dos agentes infecciosos sospechosos de haber ocasionado el cuadro clínico, *Leptospira* spp. y *Rickettsia* spp., a partir de ADN obtenido de los tejidos extraídos en la necropsia del paciente (corazón, hígado, pulmón, riñón y líquido pericárdico) y de los roedores domiciliarios (riñón). Para la extracción de ADN de los tejidos, se utilizó el estuche comercial Wizard™ (Promega, USA) en 30 mg de los tejidos y un volumen de líquido pericárdico de 200 µl. La integridad y pureza del ADN se analizaron empleando un equipo NanoDrop 2000™ (Thermo Scientific, USA) mediante electroforesis en gel de agarosa al 1% y 90 V durante 40 minutos.

Para el diagnóstico de rickettsiosis, las muestras de ADN se procesaron mediante reacción en cadena de la polimerasa cuantitativa (qPCR), empleando una sonda Taqman específica para la detección del gen *gltA* (citrato sintasa) de bacterias del género *Rickettsia* del grupo de las fiebres manchadas. Además, se usó PCR convencional para los genes *gltA* y *ompA* (10), cuyo resultado fue positivo en corazón, pulmón, hígado, riñón y suero.

Para el diagnóstico de leptospirosis, se hizo una PCR de punto final empleando el gen *rrs 16S*, tomando como poder de discriminación de la especie un tamaño de 331 pb (11). En la PCR de punto final, se empleó el gen *rrs 16S*, fijando el poder de discriminación para la especie en un tamaño de 331 pb (11). La PCR en tiempo real se hizo en un termociclador LightCycler 96 System™ (Roche, Suiza) con el siguiente ciclo de amplificación: preincubación a 95 °C durante 60 segundos y tres ciclos de amplificación seguidos de desnaturalización inicial a 95 °C durante 20 segundos, temperatura de acoplamiento de 54 °C durante 30 segundos y de 72 °C durante 20 segundos, y 60 ciclos. Las concentraciones de los reactivos utilizados para la PCR en tiempo real fueron: 0,5 µl de cada cebador (0,25 µM), 10 ul de mezcla maestra (1X) (Qiagen Type-it HRM™), 7 µl de agua para PCR y 1 µl de ADN (200 ng/µl), para un volumen final de 20 µl en cada reacción. El resultado fue positivo para la muestra de suero.

## Tipificación multilocus de secuencias para la genotipificación de *Leptospira* spp.

Los alelos para la tipificación *multilocus* de secuencias (*Multilocus Sequence Typing,* MLST) provenían del esquema propuesto por Boonsilp, *et al*. (12), y se emplearon las enzimas ribocinasa (pfkB), descarboxilasa 2- oxoglutarato deshidrogenasa (sucA), triosa fosfato isomerasa (tpiA), transferasa Acyl-CoA (caiB), el compuesto UDP N-acetil glucosamina fosforilasa (glmU), el determinante de la proteína rodA (mreA), y la subunidad α NAD (P) deshidrogenasa (pntA). Las secuencias obtenidas de los genes amplificados se analizaron según el esquema registrado en la base de datos en https://pubmlst.org/leptospira/, con base en el cual se definió la secuencia tipo (ST) de *Leptospira* spp. (13).

## Análisis filogenético de secuencias

Todos los productos de amplificación se secuenciaron en Macrogen™ (Seúl, Corea) con el método de secuenciación ILUMINA y las secuencias se analizaron con el programa MEGA7. El alineamiento de secuencias se hizo empleando el programa Clustal W a partir de un análisis comparativo con las secuencias de consenso publicadas en el *National Center for Biotechnology Information* (NCBI). Para la construcción de los árboles filogenéticos, se infirieron las relaciones evolutivas mediante el método de unión de vecinos (*neighbor-joining*) y las distancias evolutivas se calcularon con el método de Kimura 2.

El análisis molecular de la muestra de suero del paciente fallecido confirmó la presencia de *L. interrogans* serovar Copenhageni ST78, y la presencia de *R. ricketsii* en las muestras de tejidos y suero, en tanto que, en las muestras de riñón de los roedores capturados en el domicilio del paciente, también se identificó el mismo serovar y el ST6.

## Consideraciones éticas

La familia del paciente fallecido autorizó la publicación del caso.

## Discusión

En el presente caso fatal de síndrome febril con manifestaciones hemorrágicas, se demostró una infección mixta por leptospirosis y rickettsiosis mediante diagnóstico molecular *post mortem*. En los resultados de la tipificación molecular empleando MLST de *Leptospira* spp., se encontró la secuencia ST78 correspondiente al serovar Copenhageni, la misma que se identificó en un roedor capturado en el domicilio del paciente; también, se identificó *R. rickettsii* mediante análisis filogenético y PCR empleando los genes *gltA* y *ompA*. 

La región del noroccidente de Colombia es una zona endemo-epidémica para leptospirosis en humanos (11) y endémica para rickettsiosis (14). Estas dos enfermedades zoonóticas son reconocidas en la zona de Urabá y se relacionan con la trasmisión de agentes etiológicos por la picadura de garrapatas infectadas con rickettsias y por contacto directo con la orina de animales reservorios de leptospiras o, indirectamente, con ambientes o aguas contaminadas con el microrganismo (13).

La presentación clínica de este caso y su desenlace tienen una condición especial, considerando los mecanismos descritos en la epidemiología de las dos enfermedades. El paciente relató un episodio de contacto estrecho con un entorno potencialmente infeccioso antes de iniciar el cuadro clínico febril, cuando hizo la limpieza del alcantarillado de las bananeras donde detectó la presencia de abundantes ratas, reconocidas como los principales reservorios del microrganismo responsable de la leptospirosis humana (15).

Esta condición puede explicar la presentación de un cuadro clínico inicial de leptospirosis en la primera mitad de evolución del caso. Tratándose de un paciente residente en un área endémica para leptospirosis, era factible considerar que el inóculo bacteriano fue masivo, dado el estado de los alcantarillados; no fue así y ello supuso una falta de control inmunitario de una enfermedad con un curso sintomático inicial leve e inespecífico. Se han descrito diferentes formas de leptospirosis en humanos, con variados síntomas, pero generalmente se presenta en dos fases: una septicémica, primero, y luego una fase inmune. En las formas graves de la enfermedad, se puede presentar el compromiso de varios órganos, con falla renal, disfunción hepática, daño vascular, hemorragia pulmonar y lesiones musculares (16).

En la evolución de este caso se pudo observar que el paciente presentó inicialmente un estado febril diagnosticado clínicamente como una virosis indiferenciada del que no se recuperó. Esta condición es coherente con la evolución clínica de la leptospirosis en la fase inicial de la enfermedad. Posteriormente, a partir del noveno día de la consulta inicial, se estableció un posible contacto del paciente con garrapatas infectadas con rickettsias. Durante esos días, el paciente no acudió a consulta y retornó en el día 22 con signos más graves, como cefalea intensa, dolor articular general y fiebre. En ese momento, la sintomatología era más aguda, lo que se explica por la infección bacteriana de rickettsias en las células endoteliales y los daños microvasculares sistémicos.

El periodo de incubación reportado para rickettsiosis SFG (*Spotted Fever Group* del grupo de las fiebres manchadas) fluctúa entre 4 y 10 días (17), lo que coincide con las condiciones del paciente presentado, quien estuvo expuesto a *R. rickettsii* después del noveno día de evolución del cuadro clínico febril, cuando la sintomatología se hizo más grave.

En esta misma región hay reportes epidemiológicos de casos graves de rickettsiosis (18), leptospirosis y otras enfermedades, algunas de las cuales se presentan simultáneamente (19).

El caso que aquí se reporta es una muestra más de que la región de Urabá (Colombia) debe considerarse una zona potencialmente endémica para estas dos enfermedades y dar pie a una alerta epidemiológica dirigida al cuerpo médico de la región, de manera que se considere la inclusión de estas dos condiciones como diagnósticos diferenciales, pues la tipificación molecular llevada a cabo en este estudio demuestra la circulación de cepas consideradas muy patógenas.

El inicio del tratamiento antimicrobiano en una fase muy avanzada de las manifestaciones clínicas, podría explicarse por el hecho de que la malaria y el dengue son enfermedades de relevancia en la región de Urabá y ambas se asocian con síndromes febriles agudos, por lo cual, en ocasiones, no se considera el tratamiento antimicrobiano sino hasta después de confirmar una infección bacteriana.

Se sabe que, en estas dos enfermedades, están indicados antibióticos específicos que reducen la letalidad si se suministran de manera oportuna, idealmente en los primeros cinco días de la enfermedad. En algunos estudios se ha demostrado que la resolución de la enfermedad obedece a una intervención terapéutica rápida, incluso en presentaciones atípicas (20,21).

En el presente caso, el retardo en administrar el tratamiento antibiótico le restó eficacia, debido al ya evidente deterioro clínico con falla multiorgánica: la gentamicina y la nitrofurantoína se iniciaron tres días antes del deceso y, la ceftriazona, la doxiciclina y la penicilina sódica, un día antes. El hecho de que en la región no haya acceso al diagnóstico molecular confirmatorio de ninguna de estas dos enfermedades, lo que habría orientado al personal de salud en cuanto al tratamiento del paciente, impidió cambiar el curso de su condición.

Otro de los agravantes en este caso fue la respuesta inmunitaria exacerbada, descrita recientemente en infecciones como la rickettsiosis y la leptospirosis. Esta se ha relacionado con una producción descontrolada de citocinas (“tormenta de citocinas”) que se presenta en las formas graves de estas infecciones, a la que sigue un estado de inactividad del sistema inmunológico, el cual se relaciona con sepsis y falla multiorgánica (22,23).

La fiebre manchada de las Montañas Rocosas (*Rocky Mountain Spotted Fever*) y la leptospirosis siguen siendo enfermedades infecciosas más letales asociadas, no solamente con la ausencia de sospecha diagnóstica y la administración retardada del tratamiento antibiótico con doxiciclina, sino también, con las características genotípicas de *R. rickettsii* y *L. interrogans.* Esto, aunado a los respectivos factores de virulencia, puede desempeñar un papel importante en el incremento de la tasa de mortalidad de cada una de estas enfermedades por separado y, más aún, cuando se presentan simultáneamente.

La naturaleza clínica de los síntomas, que no se diferencian de los de otras enfermedades tropicales que cursan con síndrome febril agudo, como la malaria y el dengue, limita las posibilidades del oportuno diagnóstico específico de otros procesos patológicos, como las infecciones bacterianas descritas en este caso. Se hace necesario mejorar la vigilancia epidemiológica y los métodos de diagnóstico, y generar una alerta epidemiológica inmediata que establezca como prioridad la vigilancia activa de estas enfermedades zoonóticas, además de agregar estos dos agentes infecciosos al listado de diagnósticos diferenciales del síndrome febril no malárico.
